# Effect of photobiomodulation on fatigue-related errors in ankle joint position reproduction: the moderating role of perceived ankle instability in a randomized, placebo-controlled trial

**DOI:** 10.1007/s10103-026-04966-6

**Published:** 2026-07-24

**Authors:** Yasunari Ikuta, Ryuya Yamakawa, Minoru Toriyama, Hajime Ito, Tomoyuki Nakasa, Yukio Mikami, Nobuo Adachi

**Affiliations:** 1 Department of Orthopaedic Surgery, Hiroshima University Hospital, Hiroshima, Japan; 2 Sports Medical Center, Hiroshima University Hospital, Hiroshima, Japan; 3 Department of Artificial Joints and Biomaterials, Graduate School of Biomedical and Health Sciences, Hiroshima University, Hiroshima, Japan; 4 Department of Rehabilitation Medicine, Hiroshima University Hospital, Hiroshima, Japan

**Keywords:** Photobiomodulation, Low-level laser therapy, Muscle fatigue, Cumberland Ankle Instability Tool, Joint position reproduction, Proprioception

## Abstract

To investigate whether pre-exercise photobiomodulation (PBM) attenuates fatigue-induced decline in ankle joint position reproduction (JPR) and determine whether perceived ankle instability modifies this response. In this randomized, placebo-controlled trial with participant blinding and blinded outcome assessment, 30 healthy young adults (age: 24.3 ± 3.7 years; 15 male, 15 female) received either PBM or placebo treatment applied to the lower-leg musculature before a fatiguing protocol. PBM parameters included a wavelength of 830 nm, average power of 1 W, and total energy of 300 J per limb. Ankle JPR was assessed before and after fatigue. Participants were stratified by Cumberland Ankle Instability Tool (CAIT) scores. All physical assessments were performed by independent outcome assessors blinded to group allocation, with each participant evaluated by the same designated assessor throughout the study. The primary analysis revealed no overall between-group difference in fatigue-related changes in ankle JPR. A secondary exploratory group × CAIT interaction was observed, suggesting potential effect modification by perceived ankle instability. In the higher perceived instability subgroup, the estimated between-group difference was consistent with less post-fatigue worsening of JPR error following PBM; however, this estimate was based on small subgroup sizes and was not adjusted for multiple comparisons. The primary hypothesis was not supported; pre-exercise PBM showed no overall effect on fatigue-induced changes in ankle JPR. The exploratory group × CAIT interaction suggests that perceived ankle instability may be an effect modifier the response to PBM. However, this hypothesis requires confirmation in a larger, preregistered trial prospectively powered for interaction testing and incorporating functional outcomes in populations with clinically diagnosed chronic ankle instability.

**Supplementary Information:**

The online version contains supplementary material available at 10.1007/s10103-026-04966-6.

## Introduction

Lateral ankle sprains are among the most prevalent musculoskeletal injuries in sports [1, 2] and frequently progress to chronic ankle instability (CAI) [1, 3]. CAI is characterized by recurrent sprains and episodes of giving way [4] and represents a multifactorial condition involving both mechanical and functional impairments. Mechanical instability includes structural and arthrokinematic deficits, such as ligamentous laxity, altered joint kinematics, and arthrokinematic restrictions, whereas functional instability is closely related to sensorimotor deficits. In addition to perceived instability, CAI is associated with multiple sensorimotor and biomechanical alterations, including prolonged peroneal muscle latency, impaired dynamic postural stability, and altered lower-limb kinematics during landing and cutting tasks [4]. Within this broader framework, proprioception encompasses the perception of joint position and movement, and ankle joint position sense (JPS), commonly assessed via joint position reproduction (JPR), is a component of proprioceptive function that supports accurate foot placement and neuromuscular control during dynamic tasks [5, 6]. Importantly, sport-specific movements are often performed under conditions of cumulative physical exertion rather than at rest [7]. Experimental studies indicate that localized muscle fatigue impairs ankle JPR accuracy and disrupts sensorimotor performance [8, 9], suggesting that fatigue may exacerbate proprioceptive deficits in individuals with ankle instability [4, 9].

Photobiomodulation (PBM), formerly referred to as low-level laser therapy (LLLT), is a noninvasive modality that has shown potential in attenuating exercise-related muscle fatigue. Randomized, placebo-controlled trials have reported that PBM administered prior to exercise can delay fatigue onset and improve performance [10–14]. However, these effects are highly dependent upon irradiation parameters, experimental conditions, and exercise models, with some studies reporting no improvement in exercise performance or physiological responses following PBM [15]. In the lower limbs, PBM has been reported to attenuate fatigue-related impairments in muscles related to ankle function, including plantar flexors [16].

Despite accumulating evidence on PBM and muscle fatigue, whether PBM attenuates fatigue-induced impairments in ankle JPS remains largely unexplored. This gap is clinically relevant because proprioceptive control during fatigue has been associated with neuromuscular performance and injury risk during athletic activity [17]. Moreover, the acute response to PBM may depend on baseline sensorimotor status. Accordingly, individuals with greater perceived ankle instability may experience more pronounced fatigue-related proprioceptive decline and may therefore exhibit distinct responses to PBM. However, this potential moderating role of baseline instability has not been adequately examined in the context of fatigue-mediated changes in ankle JPR.

Therefore, the primary aim of this randomized, placebo-controlled, parallel-group trial, employing participant blinding and blinded outcome assessment, was to determine whether PBM applied to the lower-leg musculature attenuates fatigue-related increases in ankle JPR absolute error (AE). A secondary aim was to investigate whether baseline perceived ankle instability, quantified using the Cumberland Ankle Instability Tool (CAIT), modifies the effect of PBM on post-fatigue JPR error. We hypothesized that PBM would mitigate fatigue-related increases in AE, and that this effect would be more pronounced among individuals with perceived ankle instability.

## Methods

### Study design and ethics

This was a randomized, placebo-controlled, parallel-group trial with participant blinding and blinded outcome assessment. Participants were randomly assigned to either the active PBM group or the placebo group. In the placebo condition, the laser probe was applied to the same anatomical landmarks for an identical duration but without active laser emission. This trial was conducted at Hiroshima University Hospital between June 2024 and September 2025. Randomization was performed at the participant level, with the assigned intervention applied consistently to both lower limbs.

The study protocol was approved by the Hiroshima University Certified Review Board (approval no. CRB2023-0007), and all the experimental procedures were performed in accordance with the ethical principles outlined in the Declaration of Helsinki. Prior to enrollment, all participants provided written informed consent after receiving a detailed explanation of the study’s objectives and potential risks. This study was registered at Japan Registry of Clinical Trials (TRN: jRCTs062230104, Registration date: 7 March 2024).

### Participants

Healthy volunteers were recruited for this study. Eligible participants were adults aged 18–65 years who could comprehend the experimental procedures and provide written informed consent. To ensure a homogeneous study population and participant safety, individuals were excluded based on the following criteria: (1) a history of musculoskeletal injuries or disorders of the lower limbs or ankles requiring orthopedic intervention, including fractures, tendon ruptures, prior surgery involving implants, or other conditions that could alter ankle–foot anatomy (participants with a history of minor ankle sprains that did not necessitate medical consultation were not excluded); (2) clinical contraindications to laser irradiation, such as seizure or convulsive disorders, the presence of cardiac pacemakers or other implanted electronic devices, and dermatological conditions at the target irradiation sites; (3) a history of serious adverse reactions to PBM or LLLT; and (4) pregnancy, suspected pregnancy, or plans to become pregnant during the study period.

Footedness, which defined the dominant limb, was assessed using the Waterloo Footedness Questionnaire [18]. Subjective ankle instability was quantified using the CAIT [19], which was administered to both ankles. The minimum CAIT score across the two ankles (Minimum CAIT) was used as a participant-level indicator of the greatest degree of perceived ankle instability. This approach was selected because randomization and intervention were performed at the participant level with bilateral application of the assigned intervention, and because averaging the two ankle scores might fail to capture greater perceived instability in one ankle. The CAIT was used to quantify perceived ankle instability in the healthy volunteers rather than to establish a clinical diagnosis. Although individuals with ankle injuries requiring orthopedic treatment were excluded, participants were not systematically screened according to all International Ankle Consortium (IAC) criteria for CAI, such as the timing of the initial ankle sprain or the frequency of giving-way episodes.

**Fig. 1:**
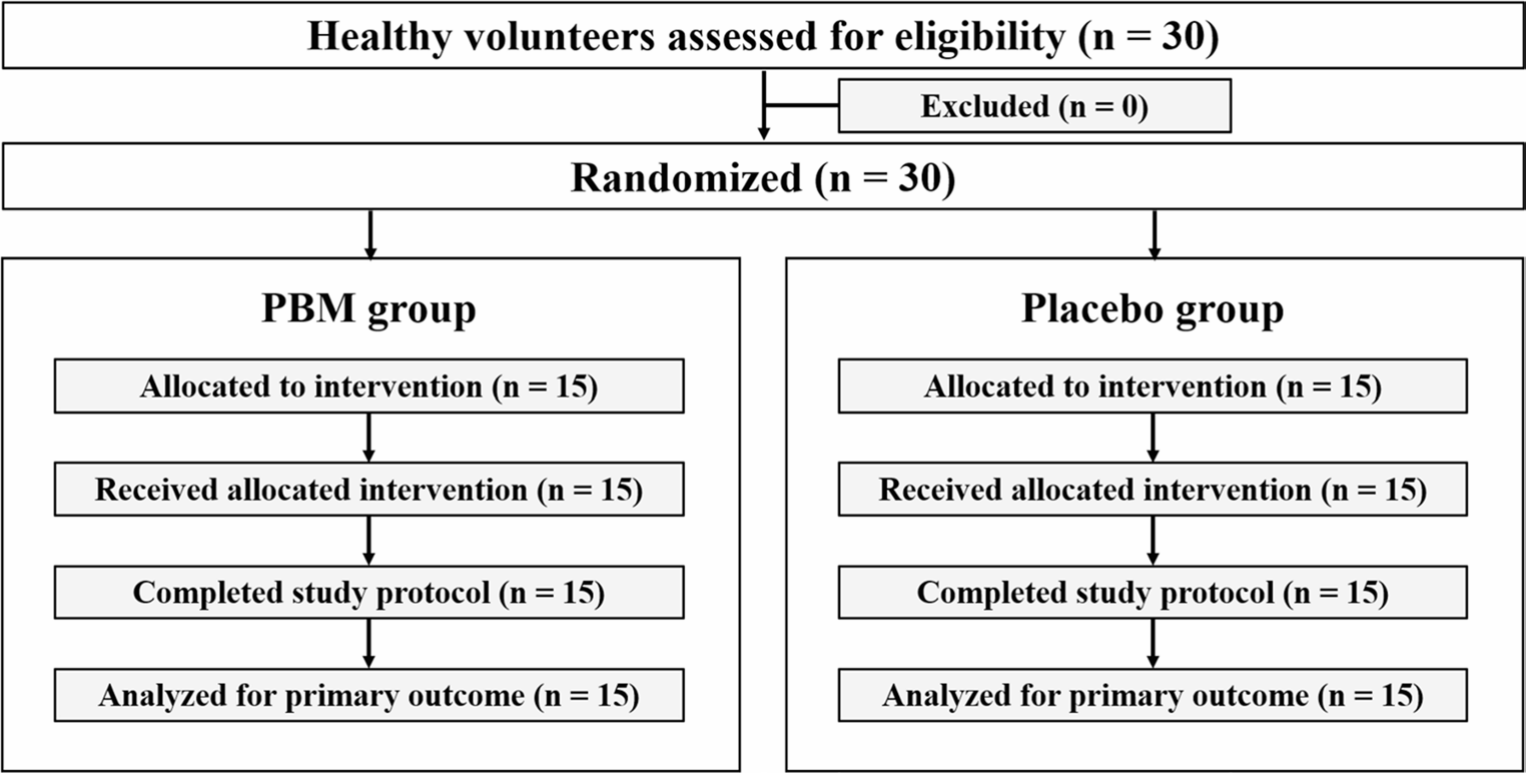
Participant flow chart. Thirty healthy volunteers were assessed for eligibility, and none were excluded. All 30 participants were randomized, received the allocated intervention, completed the study protocol, and were included in the primary analysis

In total, 30 healthy volunteers (15 male, 15 female; mean age 24.3 ± 3.7 years; range 21–38 years) were enrolled. Following the baseline assessment, participants were randomized in a 1:1 ratio into either the PBM group (*n* = 15) or the placebo group (*n* = 15) (Fig. 1). Table 1 summarizes the demographic characteristics and baseline CAIT scores. No significant differences were observed between the groups at the study onset.

**Table 1:** Participant characteristics

Characteristic	PBM (*n* = 15)	Placebo (*n* = 15)	*p*-value
Age, years	24.2 ± 3.9	24.4 ± 3.7	0.89
Sex, n (male/female)	8/7	7/8	1.000†
BMI, kg/m²	21.1 ± 3.0	21.5 ± 2.7	0.70
Dominant leg, n (right/left)	15/0	13/2	0.48†
CAIT (dominant), score	26.9 ± 5.3	27.5 ± 3.3	0.75
CAIT (non-dominant), score	26.9 ± 3.9	27.3 ± 2.8	0.71
Minimum CAIT, score	26.2 ± 5.6	26.7 ± 3.4	0.76

Continuous variables were compared using Welch’s t-test and categorical variables were compared using Fisher’s exact test. The minimum CAIT score was defined as the lower value for the two feet

*Abbreviations*: *BMI *Body mass index, *CAIT *Cumberland Ankle Instability Tool

### Randomization and blinding

Participants were randomized in a 1:1 ratio to either the PBM or placebo group using a web-based randomization module within the Research Electronic Data Capture (REDCap) system. Allocation concealment was maintained, and the randomization sequence was generated and stored securely in REDCap, which was inaccessible to the investigators responsible for outcome assessment. The system disclosed the group assignment only after the participant had been formally registered by the allocator. The allocation process was managed by a designated staff member who was not involved in the acquisition of outcome measurements. Participants were blinded to group assignment. All physical assessments were performed by independent outcome assessors who were blinded to group allocation, and each participant was evaluated by the same designated assessor throughout the study.

### Experimental procedure

The experimental protocol was conducted separately for the dominant and non-dominant limbs. The dominant limb was consistently assessed first, followed by the non-dominant limb, with a 10-min rest period between the sides to minimize carryover effects. This fixed sequence was strictly adhered to ensure standardization across all participants.

For each limb, participants first completed a standardized warm-up on an isokinetic dynamometer (Biodex System 4; Biodex Medical Systems, Shirley, NY, USA), followed by baseline isokinetic strength testing with simultaneous surface electromyography (EMG) recordings. The assigned intervention (active PBM or placebo) was then administered to the target lower leg muscles. Immediately following the intervention, perceived exertion and fatigue were recorded using the Borg Rating of Perceived Exertion (RPE) scale and the Visual Analog Scale (VAS), respectively. Baseline ankle JPR was subsequently assessed (pre-fatigue assessment).

**Fig. 2:**
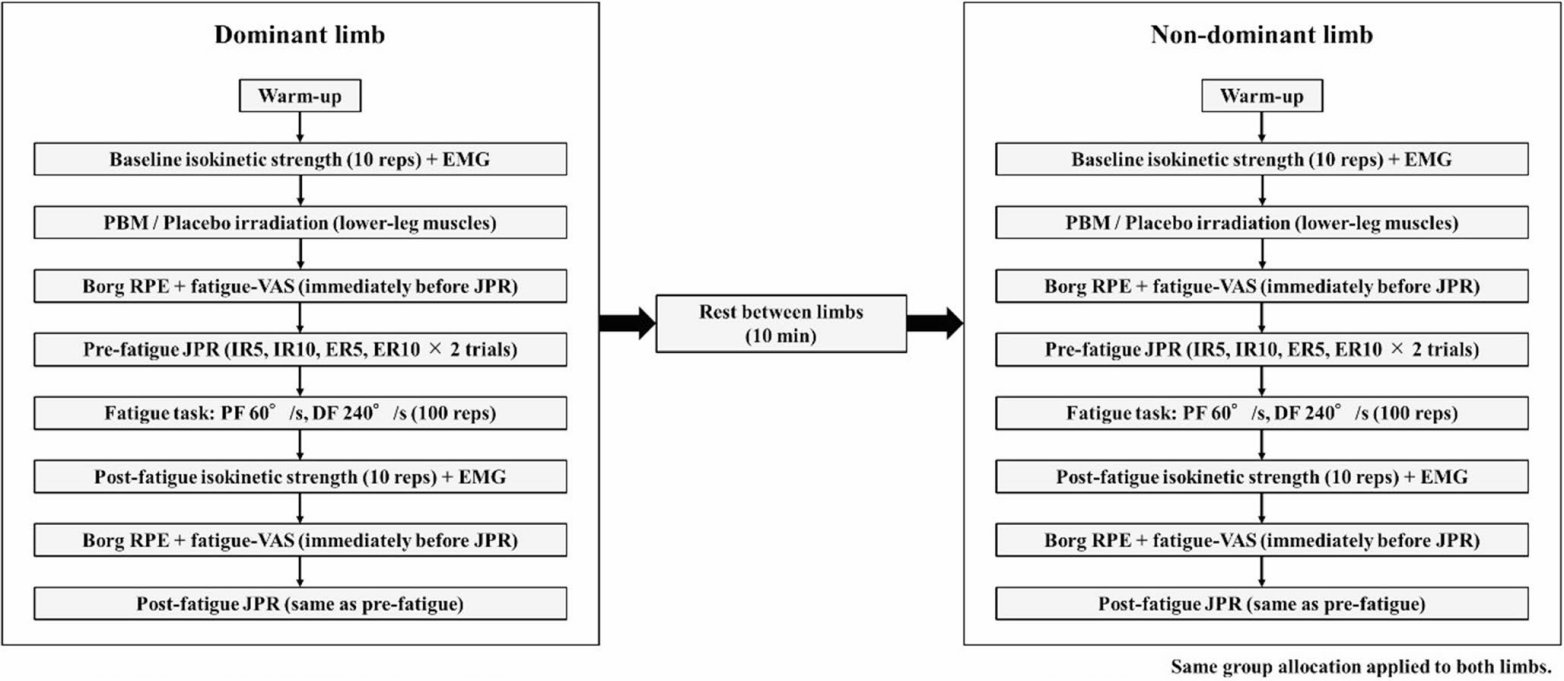
Testing was first performed on the dominant limb and then on the non-dominant limb after a 10-min rest. For each limb, baseline isokinetic strength was assessed using simultaneous surface electromyography (EMG) followed by photobiomodulation (PBM) or placebo irradiation of the lower leg muscles. The Borg RPE and fatigue VAS scores were obtained immediately before each joint position reproduction (JPR) assessment. Pre-fatigue JPR was measured before a fatigue task consisting of 100 repetitions of plantar flexion (60°/s) and dorsiflexion (240°/s) using a Biodex dynamometer. Post-fatigue strength/EMG and JPR tests were performed

Participants then completed a standardized fatigue task using a dynamometer. Immediately upon completion, isokinetic strength testing and EMG recordings were repeated, and Borg/VAS ratings were obtained. Finally, the JPR test was repeated to quantify fatigue-related changes in proprioceptive performance (post-fatigue assessment). The experimental timeline is shown in Fig. 2.

### Photobiomodulation intervention

PBM was administered using a low-power semiconductor laser system (Semiconductor Laser Sheep; UNITAC Co., Ltd., Onomichi, Japan) with a central wavelength of 830 nm and a pulsed emission profile (pulse width: 20 ms; frequency: 5 Hz). The intervention was delivered once per limb immediately prior to the pre-fatigue assessment. Irradiation was performed in contact mode with minimal pressure to ensure that the probe was maintained perpendicular to the skin surface. The probe featured a spot diameter of 14 mm, resulting in an estimated irradiation area of 1.54 cm² per point. Based on the manufacturer’s specifications, the device was operated at an average power of 1 W (peak power, 10 W). Each anatomical point was irradiated for 15 s, delivering 15 J of energy per point, which corresponded to an energy density (fluence) of approximately 9.74 J/cm² per point.

**Fig. 3:**
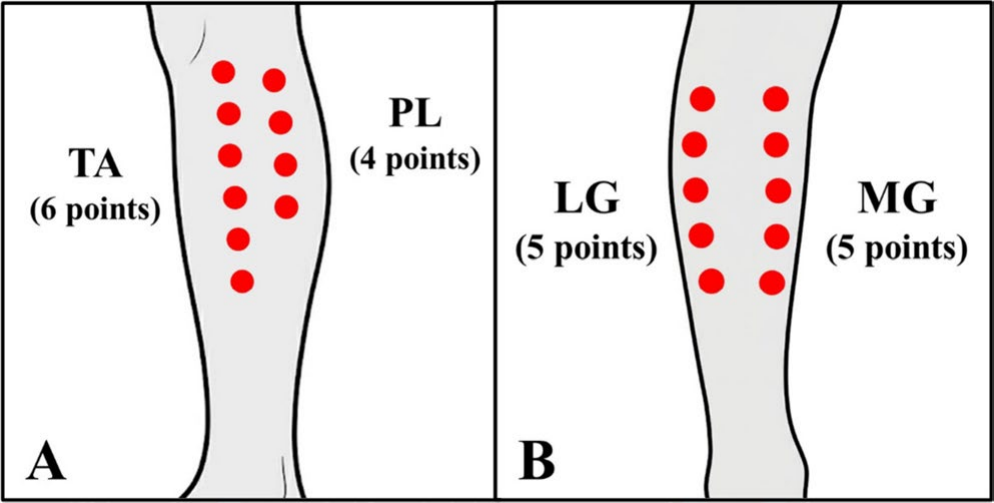
Photobiomodulation irradiation sites over the lower-leg musculature. **A** Anterolateral schematic view of the left lower leg showing irradiation sites over the tibialis anterior (TA, 6 points) and peroneus longus (PL, 4 points). **B** Posterior schematic view of the left lower leg showing irradiation sites over the lateral gastrocnemius (LG, 5 points) and medial gastrocnemius (MG, 5 points). A total of 20 irradiation points were applied per limb

The irradiation protocol targeted the following lower leg musculature per limb: the tibialis anterior (TA; 6 points), peroneus longus (PL; 4 points), and medial and lateral heads of the gastrocnemius (MG and LG; 5 points each) (Fig. 3). This comprehensive approach involved 20 irradiation points per limb, culminating in a total energy delivery of 300 J. The PL was selected as the representative lateral ankle stabilizer because its more superficial anatomical location enabled more consistent probe placement and irradiation than the deeper peroneus brevis. The peroneus brevis was not separately targeted because the total irradiation time was standardized to 5 min per limb, with 20 predefined irradiation points distributed across the TA, PL, MG, and LG. For the placebo condition, an identical device and contact procedure were employed. The probe was applied to the same anatomical landmarks for the same duration (15 s per point) as in the active PBM group; however, the system was configured such that no laser energy was emitted.

### Fatigue task

To minimize the risk of muscle strain, all participants performed a series of standardized stretching exercises targeting the musculature of the primary lower leg before testing. The fatigue protocol comprised 100 consecutive maximal repetitions of ankle plantar flexion and dorsiflexion executed on a Biodex System 4 dynamometer. The device was operated in the concentric mode with constant angular velocities of 60°/s for plantar flexion and 240°/s for dorsiflexion. The higher angular velocity for dorsiflexion was selected based on preliminary protocol testing, which showed earlier decline in dorsiflexor torque than plantarflexor torque, making completion of the 100-repetition task difficult under comparable velocity conditions. Accordingly, dorsiflexion was performed at 240°/s to reduce the mechanical demand per repetition and facilitate completion of the standardized fatigue protocol. A concentric-only fatigue protocol was selected to standardize angular velocity and range of motion across participants and to minimize the risk of acute muscle or tendon strain during repeated maximal-effort contractions in healthy volunteers. During the task, the participants were seated with the hip flexed to approximately 85°, in accordance with the manufacturer’s guidelines. To minimize compensatory movements and ensure isolated ankle joint excursion, the trunk and thigh were stabilized with harness straps and the foot was firmly secured to the footplate using adjustable hook-and-loop fasteners. Participants were instructed to perform the task continuously without rest through their full comfortable range of motion. To maintain maximal effort, standardized verbal encouragement was provided throughout the protocol. Participants were informed that the protocol could be discontinued at any time due to pain or safety concerns; however, all participants completed the 100-repetition task without premature discontinuation.

## Outcome measurements

### Ankle joint position reproduction

**Fig. 4:**
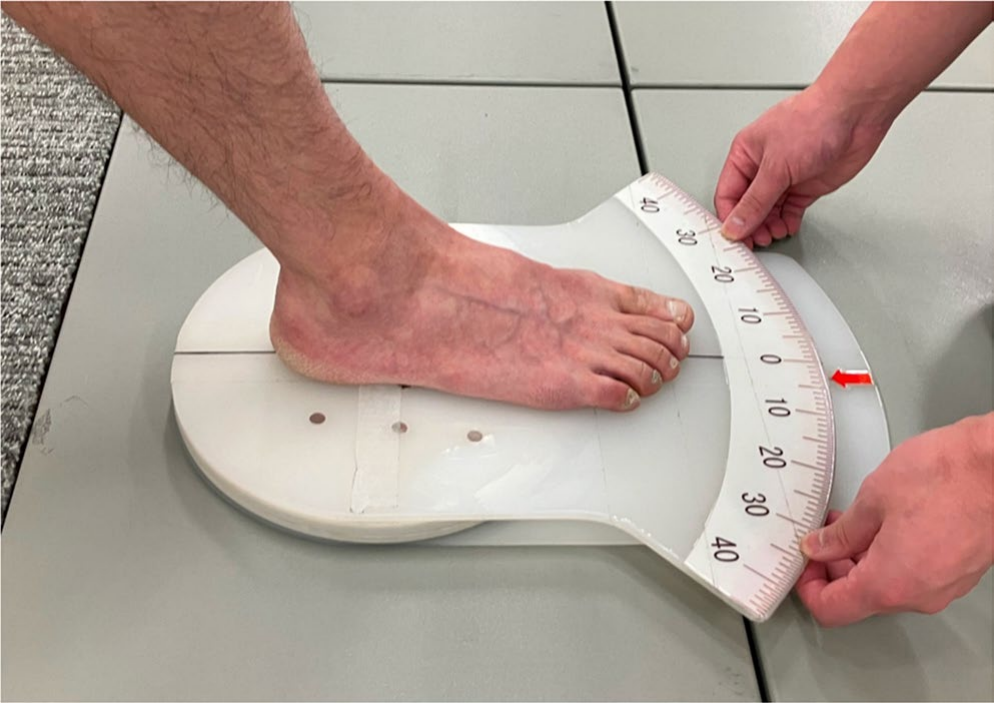
Measurement of ankle joint position reproduction using a custom goniometer footplate. The bare foot was positioned and aligned on the footplate with the ankle positioned at 20° plantar flexion. The footplate allowed controlled internal and external rotations around a pivot aligned approximately with the rotational axis of the ankle

Participants were positioned in a seated position, with their knees flexed to 70°. The participant’s bare foot was carefully positioned and aligned on a custom goniometer footplate [20] (Nakamura Brace Co., Shimane, Japan) with the ankle maintained at 20° plantar flexion (Fig. 4). The footplate mechanism facilitated internal (IR) and external (ER) rotation, with the pivot point precisely aligned with the rotational axis of the ankle; specifically, the center of rotation was positioned anteroinferior to the calcaneal tuberosity.

To eliminate visual feedback, the participants were blindfolded throughout the assessment. They were instructed to maintain complete relaxation of the lower leg musculature during the passive positioning phase. Starting from the neutral position (0°), the examiner passively rotated the footplate to one of the four target angles (IR 5°, IR 10°, ER 5°, or ER 10°) at a constant angular velocity of approximately 1°/s. Target angles of 5° and 10° were selected as mid-range joint positions are particularly relevant to muscle spindle-mediated proprioceptive feedback. This approach also ensured that all participants could safely and consistently reach these targets, especially during eversion tasks. Once the target angle was reached, the position was maintained for 5 s to allow proprioceptive encoding. The ankle was then returned to the neutral position at the same velocity, after which the participant was required to actively reproduce the target angle by rotating the footplate toward the perceived position.

The reproduced angle (response angles) was recorded for each trial. Proprioceptive accuracy was quantified using the AE, defined as the absolute difference between the target and response angles (|response − target|). Two trials were conducted for each of the four target angles in a standardized, predefined sequence. Measurements were recorded for both the dominant and non-dominant limbs before (pre) and after (post) the fatigue task. The fatigue-related change was calculated as ΔAE (post-fatigue − pre-fatigue). For the primary analysis, ΔAE values of the dominant limb were averaged across the four target angles to provide a single participant-level outcome.

### Isokinetic muscle strength

Isokinetic ankle strength was quantified for both limbs using a Biodex System 4 dynamometer under the same mode and velocities as the fatigue protocol (concentric mode: 60°/s plantar flexion and 240°/s dorsiflexion) through the participant’s full range of motion. The assessment involved 10 consecutive maximal-effort repetitions of plantar flexion and dorsiflexion per limb with standardized verbal encouragement. Peak torque (N·m) was recorded for each repetition. The mean peak torque values across 10 repetitions were divided by body mass (kg), multiplied by 100, and expressed as mean peak torque/body weight (%) for subsequent analyses.

### Surface electromyography

Surface EMG assessment was prespecified as an exploratory measure of neuromuscular activation and fatigue. EMG data were collected from all participants during isokinetic strength testing; however, these outcomes were not prespecified as primary endpoints. Signals were acquired from the TA, PL, MG, and LG using a wireless surface EMG system (Trigno; Delsys Inc., Natick, MA, USA). Following standard skin preparation, electrodes were placed over the center of each muscle belly in accordance with the SENIAM guidelines. The EMG signals were sampled at 1,000 Hz, bandpass filtered at 20–450 Hz using a fourth-order zero-lag Butterworth filter, and notch filtered at 60 Hz and its harmonics to attenuate the power-line interference.

Each isokinetic repetition was identified from the ankle joint angle signal recorded simultaneously via an analog input channel of the Trigno system, with dorsiflexion and plantarflexion peaks defined as the local maxima and minima of the angle trace, respectively. To quantify fatigue-related spectral shifts, the median frequency (MDF) was computed for each repetition and movement phase (dorsiflexion and plantar flexion, separately) from the power spectral density, estimated using Welch’s method in MATLAB (MathWorks, Natick, MA, USA). For the pre- and post-fatigue conditions, the middle eight repetitions of each 10-repetition maximal-effort isokinetic set were selected for analysis to exclude potential familiarization and terminal fatigue effects. To further explore the subgroup-specific JPR finding, a post-hoc descriptive EMG analysis was restricted to the higher perceived instability subgroup.

For data quality control, EMG recordings were excluded prior to analysis if the signal duration was insufficient to cover the full isokinetic set (i.e., the recording was truncated or nearly empty), or if data from any of the four required channels were absent. These criteria ensured that only trials that were unaffected by hardware malfunction or electrode disconnection during testing were included in the analysis. After applying these data-quality criteria, analyzable dominant-limb EMG recordings were available for 11 of the 15 participants in the higher perceived instability subgroup.

### Perceived exertion and fatigue

Subjective intensity of physical effort was quantified using the Borg RPE scale (6–20). Perceived muscle fatigue was assessed using a 100-mm VAS (fatigue-VAS), where 0 indicated “no fatigue” and 100 indicated “exhaustion.” Both measures were obtained (1) immediately after the PBM or placebo intervention and (2) immediately after the fatigue task and post-fatigue isokinetic strength testing.

### Statistical analysis

Since no prior study examined the effect of PBM on fatigue-related changes in ankle JPR, a formal a priori power calculation was not performed as no directly applicable effect-size estimate was available. This exploratory randomized trial adopted a feasibility-based target sample size of 30 participants (60 limbs), determined by the number considered feasible to recruit and assess within the planned enrollment period. The study was not powered to provide definitive evidence of efficacy or to test interaction effects. The primary outcome was the change in AE (ΔAE), calculated as post-fatigue − pre-fatigue. To avoid potential carryover effects from prior testing of the contralateral limb, the dominant limb was prespecified for the primary analysis because it was consistently assessed first. Furthermore, the dominant limb is widely recognized as the preferred limb for tasks requiring precise positioning and control during daily and sporting activities [21]. Secondary analyses were performed for the non-dominant limb and the mean across both limbs. For the initial overall between-group comparison, a single participant-level outcome was calculated by averaging the dominant limb ΔAE across the four target angles. This dominant-limb mean ΔAE was compared between the PBM and placebo groups using a two-sided Welch’s t-test. Statistical significance was set at *p* < 0.05.

To investigate whether baseline perceived ankle instability modified the effect of PBM on fatigue-induced changes in JPS, we examined the interaction between the group and CAIT scores. After obtaining CAIT scores for both ankles, the Minimum CAIT was used as the participant-level effect modifier. For the exploratory effect-modification analysis, participants were categorized as having higher (Minimum CAIT ≤ 27) or lower (Minimum CAIT ≥ 28) perceived instability. The threshold was based on the original CAIT validation cutoff of 27.5 and was used for symptom-based stratification within this healthy cohort [19]. Effect modification was evaluated using a linear mixed-effects model (LMM). The model used ΔAE as the dependent variable and included group, CAIT category, limb side (dominant vs. non-dominant), and target angle (modeled as a four-level categorical factor) as fixed effects, together with the interaction term of interest (group × CAIT category). To account for within-participant correlations, random intercepts were used for each participant. Two-sided p-values were reported for all tests.

Following confirmation of a significant group × CAIT interaction, exploratory subgroup analyses were conducted separately for each CAIT category. For these stratified comparisons, the primary analysis evaluated the dominant-limb mean ΔAE (averaged across the four target angles) using Welch’s t-tests. Additionally, as a sensitivity analysis, separate LMMs were fitted within each CAIT subgroup to assess the robustness of the findings while preserving the four target angle measurements of the dominant limb. These models included fixed effects for the group and target angles, with a random intercept for each participant. All statistical analyses were performed using JMP Student Edition 18 (SAS Institute Inc., Cary, NC, USA). No missing data were observed for the primary outcome. Although EMG assessment was prespecified as exploratory, the descriptive EMG analysis was restricted post-hoc to the higher perceived instability subgroup, with dominant-limb data analyzed for participants who met the prespecified data-quality criteria.

## Results

Data from 30 participants were analyzed (*n* = 15 per group). Table 2 illustrates the fatigue manipulation checks and isokinetic strength outcomes pre- and post-fatigue task. In the overall sample, the dominant limb mean ΔAE across the four target angles was 0.52 ± 1.03 and 0.52 ± 1.01 in the PBM and placebo groups, respectively. The between-group difference was 0.00 (95% CI, − 0.76 to 0.76; *p* > 0.99) (Table 3A).

**Table 2:** Fatigue manipulation checks (Borg and fatigue-VAS) and isokinetic strength outcomes before and after the fatigue task

Outcome	PBM (*n* = 15)	Placebo (*n* = 15)	*p*-value
Dominant limb			
Borg RPE (pre)	9.1 ± 2.4	9.3 ± 2.8	0.89
Borg RPE (post)	16.9 ± 1.8	17.3 ± 1.2	0.48
Fatigue-VAS (pre)	16.7 ± 15.0	15.5 ± 14.1	0.82
Fatigue-VAS (post)	76.1 ± 11.8	78.0 ± 10.3	0.65
Plantarflexion mean peak torque/body weight (%, pre)	97.7 ± 34.4	90.0 ± 36.5	0.56
Plantarflexion mean peak torque/body weight (%, post)	85.1 ± 29.3	84.5 ± 25.5	0.95
ΔPlantarflexion (post − pre)	-12.7 ± 33.8	-5.5 ± 20.7	0.49
Dorsiflexion mean peak torque/body weight (%, pre)	25.0 ± 12.1	27.5 ± 7.4	0.51
Dorsiflexion mean peak torque/body weight (%, post)	20.6 ± 14.8	22.7 ± 10.7	0.66
ΔDorsiflexion (post-pre)	-4.4 ± 7.6	-4.7 ± 6.0	0.88
Non-dominant limb			
Borg RPE (pre)	9.3 ± 2.1	9.0 ± 2.5	0.75
Borg RPE (post)	17.7 ± 1.4	17.4 ± 1.1	0.56
Fatigue-VAS (pre)	12.2 ± 10.0	13.8 ± 13.4	0.71
Fatigue-VAS (post)	82.5 ± 9.9	82.9 ± 7.1	0.88
Plantarflexion mean peak torque/body weight (%, pre)	87.5 ± 31.9	93.8 ± 26.4	0.56
Plantarflexion mean peak torque/body weight (%, post)	83.6 ± 23.3	88.6 ± 26.9	0.59
ΔPlantarflexion (post − pre)	-3.9 ± 17.3	-5.2 ± 17.3	0.83
Dorsiflexion mean peak torque/body weight (%, pre)	21.9 ± 9.8	27.7 ± 7.0	0.08
Dorsiflexion mean peak torque/body weight (%, post)	21.0 ± 14.1	23.7 ± 9.9	0.55
ΔDorsiflexion (post − pre)	-0.95 ± 10.0	-4.0 ± 6.0	0.32

The mean of the peak torque values across the 10 maximal-effort repetitions was divided by body mass (kg), multiplied by 100, and reported as mean peak torque/body weight (%)

Values are mean ± SD. The p-values were calculated using Welch’s t-test

*Abbreviations*: *RPE *Rating of perceived exertion, *VAS *Visual analog scale

**Table 3:** Changes in ankle joint position reproduction after the fatigue task: overall and stratified by CAIT category Panel A. Overall (*n* = 30)

Panel A. Overall (n = 30)					
Characteristic	PBM ΔAE (°)	Placebo ΔAE (°)	Difference (°)	95% CI	*p*-value
Dominant (primary)	0.52 ± 1.0	0.52 ± 1.0	0.00	−0.76, 0.76	> 0.99
Non-dominant	0.25 ± 1.0	-0.44 ± 0.9	-0.68	−1.4, 0.03	0.06
Mean of both limbs	0.38 ± 0.8	0.04 ± 0.6	-0.34	−0.85, 0.17	0.18

Panel B. Stratified by CAIT category (based on Minimum CAIT)					
B-1. Higher perceived ankle instability (Minimum CAIT ≤ 27; *n* = 15; PBM *n* = 6, Placebo *n* = 9)					
Characteristic	PBM ΔAE (°)	Placebo ΔAE (°)	Difference (°)	95% CI	*p*-value
Minimum CAIT, score	21.5 ± 6.4	24.9 ± 3.2	3.4	-3.3, 10.1	0.27
Dominant (primary)	-0.24 ± 0.64	0.81 ± 1.0	1.1	0.14, 1.96	0.027*
Non-dominant	-0.10 ± 0.74	-0.39 ± 1.03	-0.28	-1.27, 0.7	0.54
Mean of both limbs	-0.17 ± 0.58	0.21 ± 0.58	0.38	-0.29, 1.06	0.23

B-2. Lower perceived ankle instability (Minimum CAIT ≥ 28; n = 15; PBM n = 9, Placebo n = 6)					
Characteristic	PBM ΔAE (°)	Placebo ΔAE (°)	Difference (°)	95% CI	p-value
Minimum CAIT, score	29.3 ± 1.0	29.5 ± 0.84	0.17	-0.87, 1.2	0.73
Dominant (primary)	1.02 ± 0.94	0.07 ± 0.94	-0.95	-2.04, 0.14	0.08
Non-dominant	0.48 ± 1.08	-0.51 ± 0.87	-0.99	-2.09, 0.11	0.07
Mean of both limbs	0.75 ± 0.69	-0.22 ± 0.47	-0.97	-1.62, -0.32	0.0065

* The mean of both limbs was calculated for each participant by averaging the values of the dominant and non-dominant limbs. Difference = Placebo − PBM; positive values indicate greater post-fatigue worsening (larger ΔAE) in the placebo group

*AE *Absolute error, *CAIT *Cumberland Ankle Instability Tool, *CI *Confidence interval, *PBM *Photobiomodulation

However, the group × CAIT interaction was statistically significant in the LMM (F = 9.26, *p* = 0.0053), indicating that the intervention response was modulated by perceived ankle instability. Specifically, the between-group difference in fatigue-related changes in AE varied by approximately 1.35° between participants with higher perceived instability (Minimum CAIT ≤ 27) and those with lower perceived instability (Minimum CAIT ≥ 28) (interaction estimate = 1.35°, 95% CI, 0.44–2.27). In the LMM, limb side was associated with ΔAE (F = 5.99, *p* = 0.019), with the dominant limb demonstrating a larger ΔAE than the non-dominant limb (estimate = 0.61°).

The median Minimum CAIT scores were 26 (IQR, 22–27; range, 10–27) and 30 (IQR, 28–30; range, 28–30) in the higher and lower perceived instability subgroups, respectively. In the higher perceived instability subgroup (*n* = 15), the dominant-limb mean ΔAE was − 0.24 ± 0.64 in the PBM group (*n* = 6) and 0.81 ± 1.00 in the placebo group (*n* = 9). The between-group difference was 1.05 (95% CI, 0.14 to 1.96; *p* = 0.027) (Table 3B-1). Conversely, the between-group difference was − 0.28 for the non-dominant limb (95% CI, − 1.27 to 0.70; *p* = 0.54) and 0.38 for the bilateral average (95% CI, − 0.29 to 1.06; *p* = 0.23). Thus, the estimated difference observed for the dominant limb was not reproduced in the non-dominant-limb or bilateral-average analyses. Within this subgroup, exploratory angle-specific analyses showed numerically larger between-group differences at the IR targets than at the ER targets (Supplementary Material, Online Source 1).

In the lower perceived instability subgroup (*n* = 15), the between-group difference was − 0.95 for the dominant limb (95% CI, − 2.04 to 0.14; *p* = 0.082), − 0.99 for the non-dominant limb (95% CI, − 2.09 to 0.11; *p* = 0.07), and − 0.97 for the bilateral average (95% CI, − 1.62 to − 0.32; *p* = 0.0065). All three estimates were consistent with greater post-fatigue worsening in the PBM group (*n* = 9) than in the placebo group (*n* = 6), although only the bilateral-average comparison reached statistical significance (Table 3B-2).

In sensitivity analyses utilizing LMMs on the dominant limb data (preserving all four target angles), the main effect of group remained significant in the higher perceived instability subgroup (*p* = 0.040) but not the lower perceived instability subgroup (*p* = 0.077). These findings were consistent with the primary analysis based on the averaged ΔAE. Exploratory EMG MDF outcomes from the post-hoc analysis of the higher perceived instability subgroup are provided in Supplementary Material, Online Source 2. Given the subgroup-restricted, post-hoc analysis and the limited number of analyzable dominant-limb recordings after data-quality control (11/15), these findings were treated as exploratory descriptive data and were not used for mechanistic interpretation.

## Discussion

In this randomized, placebo-controlled trial, the primary hypothesis was not supported as pre-exercise PBM did not produce an overall between-group difference in fatigue-related changes in ankle JPR. A secondary exploratory group × CAIT interaction suggested that perceived ankle instability may modify the response to PBM. The estimated between-group difference in the higher perceived instability subgroup was consistent with less post-fatigue worsening of JPR error with PBM; however, this finding was based on small subgroup sizes and multiple comparisons without adjustment and should not be interpreted as evidence of a confirmed subgroup-specific treatment effect. Accordingly, this hypothesis-generating finding requires confirmation in a trial prospectively powered for interaction testing.

The limb-specific secondary analyses were not fully consistent. In the higher perceived instability subgroup, the estimated difference indicating less post-fatigue worsening with PBM was observed only for the dominant limb and was not reproduced in the non-dominant-limb or bilateral-average analyses. Conversely, in the lower perceived instability subgroup, the dominant-limb, non-dominant-limb, and bilateral-average estimates were all in the direction of greater post-fatigue worsening with PBM, although only the bilateral average reached statistical significance. Previous studies have documented limb-dominance-related asymmetries during gait and unilateral landing tasks [21, 22], supporting consideration of limb role as one possible contributor to the observed differences. Averaging both limbs may have reduced within-participant measurement variability and increased estimate precision. However, given the small subgroup sizes, absence of multiplicity adjustment, fixed dominant-first testing sequence, and negative ΔAE values observed in some placebo participants, these differences in statistical significance may be attributable to random variation, limb- or order-related effects, familiarization [23], or regression to the mean [24] rather than a true AE of PBM. Therefore, the bilateral-average analysis and other limb-specific subgroup results should be considered exploratory.

The fatigue protocol and measurement timing were standardized, and subjective ratings (Borg RPE and fatigue-VAS), together with dynamometer-derived indices, suggested broadly comparable fatigue induction between the groups. However, the present study was not designed to determine the mechanism underlying the exploratory group × CAIT interaction. Given that PBM was applied to the lower-leg musculature, one possible explanation is that it influenced peripheral muscle-derived proprioceptive input during fatigue [5, 25, 26]. Nevertheless, muscle spindle function, afferent signaling, metabolic responses, and central sensorimotor processing were not directly measured. Therefore, this explanation remains hypothetical, and the present findings do not establish a mechanism for the observed subgroup pattern.

From a functional perspective, preserving proprioceptive acuity during fatigue is crucial for athletic tasks that require rapid and precise sensorimotor control [6]. Although the dominant-limb between-group difference in the higher perceived instability subgroup was approximately 1°, its clinical significance remains uncertain because a formal minimal clinically important difference for ankle JPR has not been established. As a reference point, Nakasa et al. reported a side-to-side difference in inversion angle replication error of approximately 1.0° in chronically unstable ankles compared with 0.2° in healthy controls [20]. However, since the present study evaluated the between-group difference in the pre- to post-fatigue change in AE during IR and ER reproduction, these values are not directly comparable. Given that the replication error did not correlate with conventional measures of mechanical instability, small discrepancies may represent clinically relevant sensorimotor impairment independent of ligamentous laxity. Moreover, as our footplate paradigm quantified error across a single axis of motion, the observed difference of approximately 1° should be interpreted as a planar projection of an intrinsically three-dimensional (3D), coupled ankle–foot orientation error, which may underestimate true functional 3D deviations. Accordingly, the approximately 1° between-group difference should be interpreted as a potentially relevant exploratory signal rather than as an established clinically meaningful effect. Within the higher perceived instability subgroup, exploratory angle-specific analysis further suggested numerically larger between-group differences at the IR targets than at the ER targets. This pattern may be relevant because IR positions approach ankle supination ranges that have been typically implicated in lateral ankle sprain mechanisms [1, 4]. Although the present study did not assess injury incidence and therefore cannot support preventive inferences, the preferential pattern at IR targets may provide a basis for future research aimed at optimizing proprioceptive control under demanding conditions [27].

This study had some limitations. First, ankle JPR was assessed in a non-weight-bearing, open-chain position using a custom goniometer footplate that quantified error in a single degree of freedom. Although this approach allowed standardized measurement and may be feasible for clinical screening, it does not fully capture the dynamic, closed-chain mechanics or 3D coupled motion of the foot–ankle complex during functional movements. Thus, the present findings may underestimate multi-planar positioning errors and should not be directly extrapolated to sport-specific tasks such as landing or cutting. This limitation should be addressed by combining custom JPR devices with 3D motion capture and force-plate analysis to quantify six degrees of freedom ankle–foot orientation errors during weight-bearing tasks.

Second, the fatigue and JPR testing protocols may not fully reproduce sport-specific ankle instability conditions. The fatigue task consisted of repeated concentric contractions; although this approach was selected for participant safety and measurement standardization, it does not replicate the eccentric muscle failure or rapid eccentric loading that may occur during lateral ankle sprain mechanisms. In addition, plantar flexion and dorsiflexion were performed at different angular velocities (60°/s and 240°/s, respectively) to account for the earlier decline in dorsiflexor torque observed during preliminary testing and to facilitate completion of the 100-repetition protocol. Nevertheless, this difference may have produced non-equivalent loading and fatigue responses between the plantarflexor and dorsiflexor muscle groups. The JPR target angles were also restricted to relatively small angles (5° and 10°), which may not fully capture proprioceptive demands near the functional end range of ankle motion. Furthermore, although averaging ΔAE across target angles improved interpretability for the primary outcome, it may have obscured angle-specific responses. Further work should incorporate eccentric or plyometric fatigue tasks and target angles closer to each participant’s functional limits while maintaining safety and measurement reliability.

Third, the study population consisted of healthy young adults stratified by CAIT-defined perceived ankle instability; therefore, the higher perceived instability subgroup should not be considered equivalent to a clinically diagnosed CAI population. Although individuals with injuries requiring orthopedic treatment were excluded, comprehensive clinical screening based on all IAC criteria was not performed. Accordingly, the present findings should be interpreted as applying to healthy individuals with perceived ankle instability, warranting caution when extrapolating these results to populations with clinically confirmed CAI. In addition, Minimum CAIT was used as a participant-level effect modifier and was not matched to the JPR outcome of each individual limb; therefore, limb-specific relationships between perceived instability and PBM response could not be determined.

Fourth, the interpretation of the interaction and subgroup findings is limited by both the study design and statistical considerations. Some participants in the placebo group showed reduced JPR error after the fatigue task, suggesting a possible learning or familiarization effect, and the absence of a time-matched no-fatigue control group prevented us from fully separating fatigue effects from practice-related changes. In addition, the group × CAIT interaction and subsequent subgroup analyses were secondary, and the sample size was not determined for interaction testing. The CAIT-stratified subgroups were small, and no multiplicity adjustment was applied to the subgroup or angle-specific comparisons. Consequently, these estimates have limited precision; therefore, the interaction, subgroup, and angle-specific findings should be considered exploratory and hypothesis-generating.

Lastly, the mechanistic interpretation of the present findings remains limited. Although EMG assessment was prespecified as an exploratory measure and data were collected from all participants, the descriptive EMG analysis was restricted post-hoc to the higher perceived instability subgroup to further explore the subgroup-specific JPR findings. After data-quality control, analyzable dominant-limb recordings were available for only 11 of the 15 participants in this subgroup. Therefore, the EMG findings were treated as exploratory descriptive data and were not used to support mechanistic conclusions. The post-hoc subgroup restriction and limited data availability also precluded a reliable assessment of associations between changes in EMG MDF and JPR outcomes. Future preregistered trials, prospectively powered to detect interaction effects, should confirm whether perceived or clinically diagnosed ankle instability moderates the effects of PBM on fatigue-related proprioceptive changes. Such studies should incorporate functional outcomes, direct mechanistic measures, dose–response assessment, durability of effects, and longitudinal follow-up.

## Conclusion

The primary hypothesis was not supported because pre-exercise PBM showed no overall effect on fatigue-induced changes in ankle JPR. A secondary exploratory interaction suggested that CAIT-defined perceived ankle instability may be a potential effect modifier of the response to PBM. This hypothesis requires confirmation in a larger, preregistered trial prospectively powered for interaction testing in clinically diagnosed CAI populations and incorporating functional outcomes. 

## Supplementary Information

Below is the link to the electronic supplementary material.

Supplementary Material 1

## Data Availability

The data that support the findings of this study are available from the corresponding author upon reasonable request.

## Declarations

### Clinical trial number

Japan Registry of Clinical Trials, TRN: jRCTs062230104, Registration date: 7 March 2024.
