# Supplementary material for: Effect of photobiomodulation on fatigue-related errors in ankle joint position reproduction: the moderating role of perceived ankle instability in a randomized, placebo-controlled trial
Source: Lasers Med Sci. 2026 Jul 24;41(1):162. doi: 10.1007/s10103-026-04966-6 (PMC13395833; doi:10.1007/s10103-026-04966-6)
Supplement: Supplementary file 1 — Supplementary Material 1 [file 10103_2026_4966_MOESM1_ESM.pdf]

Effect of photobiomodulation on fatigue-related errors in ankle joint position reproduction: the moderating role of perceived ankle instability in a randomized, placebo-controlled trial

Online Source 1. Angle-specific changes in dominant-limb ankle JPR error (ΔAE) in the higher perceived instability subgroup (exploratory)

	PBM	Placebo	Difference	95% CI	p-value
**Mean IR**	−0.21 ± 0.66	1.08 ± 1.40	1.29	0.12 to 2.47	0.034*
**Mean ER**	−0.27 ± 1.03	0.54 ± 1.77	0.81	−0.75 to 2.38	0.28

Values are presented as mean ± SD. ΔAE was calculated as post-fatigue AE minus pre-fatigue AE; positive values indicate increased error after the fatigue task. Between-group differences are expressed as Placebo − PBM. Mean IR represents the average of IR 5° and IR 10°, and mean ER represents the average of ER 5° and ER 10°. P values were calculated using two-sided Welch's t-test. This exploratory analysis was restricted to the dominant limb of the higher perceived instability subgroup (Minimum CAIT ≤ 27). $^*p < 0.05$.

Online Source 2. Electromyography (EMG) median frequency (MDF) outcomes of the dominant limb before and after fatigue in the higher perceived instability subgroup

Outcome	PBM (n=5)	Placebo (n=6)	p-value
TA (pre), Hz	96.1 ± 22.6	83.5 ± 19.5	0.36
TA (post), Hz	68.0 ± 8.6	65.6 ± 8.4	0.64
Δ (post – pre)	-28.0 ± 26.6	-18.0 ± 25.2	0.54
PL (pre), Hz	112.8 ± 37.3	112.7 ± 15.8	0.60
PL (post), Hz	100.2 ± 23.0	99.3 ± 37.4	0.96
Δ (post – pre)	-12.6 ± 28.2	-23.4 ± 25.7	0.53
MG (pre), Hz	103.6 ± 34.8	106.8 ± 17.2	0.86
MG (post), Hz	113.5 ± 24.1	102.3 ± 18.1	0.42
Δ (post – pre)	9.9 ± 18.3	-4.5 ± 13.9	0.19
LG (pre), Hz	84.3 ± 20.8	86.6 ± 14.4	0.84
LG (post), Hz	86.7 ± 13.8	107.8 ± 23.5	0.10
Δ (post – pre)	2.4 ± 9.6	21.3 ± 27.9	0.17

Values are presented as mean ± SD. MDF, median frequency (Hz); EMG, electromyography; PBM, photobiomodulation; Δ, change score (post-fatigue minus pre-fatigue). MDF was estimated from the power spectral density using Welch's method (Hamming window, 50% overlap). MDF was computed across the middle eight repetitions of the 10-repetition isokinetic set. P values were calculated using a two-sided Welch's t-test for between-group comparisons at each time point and for Δ. This exploratory EMG analysis was restricted to

the dominant limb of higher perceived instability participants with high-quality recordings (PBM, n = 5; placebo, n = 6). Exclusions were based on prespecified quality-control criteria, including insufficient signal duration (e.g., truncated or nearly empty recordings) or absence of data from any of the four required EMG channels.

Abbreviations: TA, tibialis anterior; PL, peroneus longus; MG, medial gastrocnemius; LG, lateral gastrocnemius.
